# Psychometric evaluation and cross-cultural adaptation of the Australian Pelvic Floor Questionnaire (APFQ-IR) in Iranian reproductive age women

**DOI:** 10.1038/s41598-023-50417-5

**Published:** 2023-12-27

**Authors:** Sepideh Mashayekh-Amiri, Mohammad Asghari Jafarabadi, Fatemeh Rashidi, Mojgan Mirghafourvand

**Affiliations:** 1 Students Research Committee, Midwifery Department, Faculty of Nursing and Midwifery, Tabriz University of Medical Sciences, Tabriz, Iran; 2 Cabrini Research, Cabrini Health, Melbourne, VIC 3144 Australia; 3 School of Public Health and Preventative Medicine, Faculty of Medicine, Nursing and Health Sciences, Monash University, Melbourne, VIC 3800 Australia; 4 Road Traffic Injury Research Center, Tabriz University of Medical Sciences, Tabriz, Iran; 5 Students Research Committee, Midwifery Department, Faculty of Nursing and Midwifery, Tabriz University of Medical Sciences, Tabriz, Iran; 6 Social Determinants of Health Research Center, Department of Midwifery, Faculty of Nursing and Midwifery, Tabriz University of Medical Sciences, Tabriz, Iran; 7 Medical Philosophy and History Research Center, Tabriz University of Medical Sciences, Tabriz, Iran

**Keywords:** Urological manifestations, Translational research

## Abstract

Pelvic floor disorders (PFDs), as a silent alert, is one of the pervasive debilitating health concerns among women all over the world, such that in developed countries, one in four women, suffers from PFDs. Validity and reliability of the Australian Pelvic Floor Questionnaire (APFQ) has not been determined in Iran, so to determine APFQ’s psychometric characteristics, we decided to conduct this study on women of reproductive age in Tabriz city, Iran. This methodological cross-sectional study was intended to determine the psychometric properties of the Persian version of the APFQ-IR in 5 steps including “translation process, content validity, face validity, construct validity (exploratory and confirmatory factor analyses and examination of ceiling and floor effects) and reliability” on 400 reproductive age women referring to health centers in Tabriz city, Iran, with cluster random sampling method in the period between May 2022 to September 2022. The translation process was done based on two approaches, Dual panel, and Beaton et al.’s five steps. Then, in order to evaluate content validity, face validity, and construct validity, 10 instrument and PFDs experts, 10 women from the target group investigated the instrument's items, and 400 eligible women completed the instrument. Finally, to determine the reliability, two internal consistency methods, (Cronbach's alpha and McDonald's omega) and test–retest method (ICC) were used. In the present study, content validity assessment of APFQ-IR, showed a good level of validity (CVR = 0.96, CVI = 0.94). To assess construct validity, exploratory factor analysis results on 36 items, led to the identification of 4 factors including bladder function, bowel function, prolapse symptom and sexual function, which explained 45.53% of the cumulative variance and indicated the sufficiency of the sample size (Kaiser–Meyer–Olkin = 0.750). Implementing confirmatory factor analysis, (RMSEA = 0.08, SRMR = 0.08, TLI = 0.90, CFI = 0.93, *χ*^2^/df = 3.52) confirmed the model fit indices. Finally the internal consistency and reliability was high for the entire instrument (Cronbach’s alpha = 0.85; McDonald's omega (95% CI) = 0.85 (0.83–0.87) and Intraclass Correlation Coefficient (95% CI) = 0.88 (0.74–0.94)). The Persian version of the APFQ-IR, has a good validity and reliability and has acceptable psychometric properties, thus can be used both for research purposes and for clinical evaluation of pelvic floor disorders symptoms in health centers.

## Introduction

Pelvic floor disorders (PFDs), are considered as one of the most common complaints among women refering to gynecology clinics. PFDs include a wide range of clinical manifestations that have direct effects on the urinary system, digestive system, and sexual activity. It significantly disrupts women's daily activities and quality of life (QOL). It also imposes a huge financial burden on the health care system every year^1^.

PFDs include a wide range of symptoms and anatomical changes related to the abnormal behavior of the pelvic floor muscles, classified by the range of symptoms^2^. The ranges of pelvic floor symptoms in women include lower urinary tract symptoms (LUTS) (urinary incontinence (UI), urgency and increased frequency of urination, feeling of incomplete emptying, impaired urinary elimination), bowel symptoms (fecal incontinence (FI), constipation, obstructed defecation) and sexual problems (dyspareunia, orgasm disorder), pelvic organ prolapse (POP) and genito-pelvic pain in women^3^.

The pelvic floor is an anatomic unit with a wide range of functionalities, including pelvic organs support and sexual function. Thus, it is expected that the PFDs will lead to more than one simultanous symptom, in more than one area of the pelvic floor^4^. In this regard, a review study by Vries et al. in 2022 reported that about a quarter to a third of women experienced one or more pelvic floor symptoms (such as urinary incontinence, fecal incontinence, POP) at the same time^4^. Also, the results of Kenne et al.’s study (2022) showed that at least 32 percent of women suffer from one category of PFDs. The most common of which is bowel dysfunction, with a 24.6 percent share, followed by urinary incontinence and POP, which had a 11.1 and 4.4 percent share, respectively^5^.

Generally, the prevalence of PFDs varies around the world, according to most studies, 11.5 to 35 percent of women worldwide suffer from PFDs in silence^6–8^. In this regard, the results of a study in the United States of America reported that the demand for the treatment of PFDs will increase by 35% by 2030^9^.

The main cause of PFDs is unknown. Several factors are involved in its occurrence^10^. Age^11^, ethnicity^12^, multiparity^13^, mode of delivery^14,15^, traumatic injuries^16^, history of pelvic surgery^17^, pregnancy^18,19^, vaginal delivery^20^, chronic cough^21,22^, obesity^23^, spinal disorders^24^, family history^25^, pelvic floor muscle dysfunction and genetics^25^ are the most common risk factors that can be identified.

Overally, women of reproductive age are at greater risk for PFDs^26^. Given that early treatment of PFDs allows for conservative, non-surgical treatment options such as Kegel exercises (pelvic floor muscle training (PFMT)) or pessary placement which have shown their effectiveness, therefore, in order to identify this condition early, screening tools will be very useful. The screening of this condition in health centers with a reliable instrument provides an optimal opportunity for counseling and prevention^27,28^. According to the International Consultation on Incontinence (ICI), the most reliable measure to assess the presence, severity, and impact of PFDs on the patient's activities and health is the patient-reported outcomes measures (PROMs).

PROMs are defined by the Food and Drug Administration (FDA) as "any report of health status that is provided directly by the patient, without interpretation of the patient's response by a physician or any other person"^29,30^. PROMs, an old and reliable method of data collection, are usually in the form of questionnaires that provide a clear perspective of the patient's problem, especially in multifactorial conditions, such as PFDs. Over hundreds of PROMs are available for use in the screening of pelvic floor disorders and to measure the results of treatment. Although these questionnaires were well-accepted, they often fail to address all aspects of pelvic floor dysfunction. APFQ is a simple but comprehensive tool designed to evaluate PFDs in clinical practice. Although most aspects of the pelvic floor function can be evaluated using several overlapping ICI questionnaires, completing it for patients in a typical clinical environment is very time-consuming because a single questionnaire does not cover all areas^31,32^.

APFQ, as a validated PROM for routine urogenycological evaluation and research on PFDs, designed in Australia by Baessler et al. in 2009, evaluates PFDs with 43 questions in 4 different factors (bladder function (BLF), bowel function (BF), prolapse symptom (PS) and sexual function (SF)), emphasizing the severity, degree of bother experienced and their impact on the QOL of women^33^. It has also been translated recently and used in several languages including Chinese^34^, Turkish^35^, Serbian^36^, Spanish^37^ and French^38^.

Considering the high prevalence of pelvic floor symptoms and numerous negative consequences both physically and psychologically for Iranian women, there is a great need to develop instruments for screening PFDs and use them in health centers. Since the current version of the Australian Pelvic Floor Questionnaire has not been validated in Iran and its reliability has not been investigated. Therefore, we decided to conduct this study with the aim of determining the psychometric properties of the APFQ in Iranian reproductive age women.

## Methods

### Research population and setting

This study was a methodological cross-sectional study. It was conducted on reproductive age women referring to health centers in Tabriz, Iran, from May 2022 to September 2022. The purpose of this study was to determine the psychometric properties of the Persian version of the APFQ-IR during five stages, including translation, content validity, face validity, construct validity and reliability assessment in reproductive age of women in Tabriz, Iran.

### Translation process

In order to carry out the translation process and determine the psychometric properties of the instrument, in the first step, a permission was requested from the main designers of the tool^33^. Then, to increase the accuracy of the translation process, the translation was done using two approaches, Dual panel (DP) and Beaton et al.’s five steps (Guidelines for the Process of Cross-Cultural Adaptation of Self-Report Measures). In the first approach (DP), the translation process is done in three steps^39^. The first panel (expert panel) consisted of 10 reproductive health, obstetrics and nursing education specialists. The second panel (layman panel) consisted of 10 eligible women. In the third stage (the target group panel), 400 eligible women of reproductive age completed the questionnaire in the presence of the researcher. In the second approach, according to Beaton et al.'s guidelines, the translation process was implemented through 5 stages including Stage I: Initial Translation, Stage II: Synthesis of The Translations, Stage III: Back Translation, Stage IV: Expert Committee, Stage VI: Pretesting^40^.

In the first stage, two translators (T1, T2) whose mother tongue was Farsi, performed the translation completely independently using the Forward-Translation method. The first translator was well learned in the field of PFDs and the second translator should preferably not have a medical or clinical background. This translator is called a naive translator and is more likely to recognize a different meaning of the original text than the first translator, and the translation she/he provides reflects the language used by that population and often highlights ambiguous meanings in the original questionnaire. Finally, two translators and a supervisor combined the results of the translations during sessions using the original questionnaire as well as the versions of the first translator (T1) and the second translator (T2) (production of a joint translation, T-12)^41^.

In the Backward-Translation stage, two other native English translators (BT1, BT2), who were completely blind to the original version of the questionnaire, re-translated the questionnaire into English using the T-12 version of the questionnaire. The translators also ensured that the final version of the questionnaire was comprehensible to a 12-year-old (approximately 6th grade reading level). The fourth stage is the expert committee’s review.They reviewed all the translations (T1, T2, T12, BT1, and BT2) along with the written reports from four viewpoints of Semantic Equivalence, Idiomatic Equivalence, Experiential Equivalence, and Conceptual Equivalence. The final stage is the pre-test, which seeks to use the pre-final version in the target group. In the pre-test phase, the researcher provided the final translated version of the questionnaire to 30 women of reproductive age who meet the criteria in health centers. This was done to evaluate the clarity and comprehensibility of the final version for the target group and also to examine its internal consistency. After responding, women were asked again about their understanding of the questions, the level of difficulty, and the cultural appropriateness of the phrases. Participants in this stage were encouraged to provide feedback on all sections of the questionnaire so that the final Iranian version of the questionnaire would be more culturaly acceptable in the Iranian community^41^.

### Content and face validity

After the final version of the questionnaire was prepared, in order to assess the content and face validity of AFPQ-IR, the content validity determination form was given to 10 reproductive health, obstetrics and nursing education specialists with expertise in the field of instruments and the field of PFDs and 10 eligible women. In the qualitative section, in order to measure the content validity of the AFPQ-IR, experts' opinions were received in terms of the overall structure of the questionnaire, the content of the items, Persian grammar and correct scoring and then, corrections were made. In addition, in the quantitative section, content validity ratio (CVR) and content validity index (CVI) were calculated^42^. In order to perform CVR, experts’ opinions were received about each of the items of the instrument using a 3-point Likert scale in terms of the necessity (necessary, useful but unnecessary, and unnecessary) in the instrument. After obtaining the opinions of experts in the present study, according to the analysis of content validity by using Lawshe’s CVR technique, the minimum acceptable value for 10 experts is 0.62. As a result, cases with CVR > 0.62 were kept^43^. After that, in order to determine CVI, experts were asked to determine the relevance, clarity and simplicity of the items using a 4-point Likert scale based on the Waltz and Bausell index^44^. CVI values vary between 0 and 1. Items with a CVI more than 0.79 were accepted^45^. Then, in order to determine the qualitative face validity, the items were assessed in terms of difficulty level, relevance and ambiguity by 10 eligible women (target group). In the quantitative face validity, the item impact method using a 5-point Likert scale from unimportant (1) to very important (5) was used to determine the impact score, and finally, the items with Impact score ≥ 1.5 were kept^46^.

### Construct validity

Finally, construct validity was assessed using exploratory factor analysis (EFA), with Kaiser-Meyer Olkin (KMO) and Bartlett’s test of sphericity criteria. Also, in order to determine the factors, principal component analysis method with varimax rotation (direct oblimin) was used. The amount of factor loading was considered above 0.3^47,48^. In confirmatory factor analysis (CFA), a series of indices such as the root mean square error of approximation (RMSEA < 0.08), standardized root mean square residual (SRMR < 0.10), normed Chi2 (*χ*^2^/df) < 5, comparative fit Indices including comparative fit index (CFI > 0.90) and Tucker–Lewis Index (TLI) > 0.90 were used to assess the fit of the model^49,50^. Finally, after factor analysis and removal of inappropriate items from the instrument, the floor and ceiling effect (F/C) was assessed, i.e. the samples with the highest and the lowest possible scores were judged whether the ceiling and floor effects are true about them or not, respectively.

Based on a rule of thumb, the sample size for exploratory factor analysis is classified as 50 = very poor, 100 = poor, 200 = fair, 300 = good, 500 = very good and 1000 = excellent^51^. The number of samples for construct validity assessment in factor analysis is 5 to 10 samples for each instrument item. Therefore, by considering 5 samples in each item with design effect equal to 1.5 and considering 30% attrition, 400 eligible women who had referred to Tabriz health centers were selected using cluster sampling method. For sampling, a quarter of the centers were randomly selected using the website http://www.random.org and the list of samples were selected based on SIB system (integrated health system). The inclusion criteria included all women regardless of having diagnosis of PFD or not, women of reproductive age (15–49 years), having sexual activity, monogamous husband and not having a known pregnancy at the time of the study. Women with a dementia, psychological disorders such as depression, intellectual disabilities, schizophrenia, addiction to drugs and/or alcohol, previous or current malignancy, being in 12 months after delivery, recent history of urinary tract infection (UTI), a history of gynecological surgery including reconstructive, cosmetic surgery and pelvic surgery, sexually transmitted diseases (STDs), and White Blood Cell (WBC) > 3 in the urine analysis test (U/A) and illiterate women were excluded from the study.

Then, after providing a comprehensive explanation about the research to the participants and receiving informed consent, the researcher provided them with socio-demographic and obstetric characteristics questionnaire and the Persian version of the Australian Pelvic Floor Questionnaire (APFQ-IR). Socio-demographic and obstetric characteristics included information such as age, body mass index (BMI), gravidity, parity, education level, occupation, income, smoking status, type of delivery, hysterectomy, prolapse surgery, and family history of PFDs. The APFQ questionnaire, was used to investigate PFDs. Higher scores indicate more severe pelvic floor disorders. Its validity (content validity, face validity, construct validity) and reliability were assessed in this study. This instrument was designed by Baessler et al. in Australia, and it contains 43 questions and is divided into four factors: BLF (Q1-15), BF (Q16-27), PS (Q28-32), and SF (Q33-43). The scoring is not based on the Likert scale. Most of the questions are scored from 0 to 3 using different descriptions such as Never, Occasionally, Frequently, and Daily to evaluate intensity/repetition, and Not at all, Slightly, Moderately, and Greatly to estimate bothersome symptoms. The scores in each area are calculated separately, divided by the number of questions in each field and then multiplied by 10. The overall score for each area is between 0 and 10, and the maximum score for PFDs is 40^52^. The higher the score, the more intense the PFDS.

### Reliability

On the other hand, to determine the reliability of the questionnaire, test–retest reliability and internal consistency were used^53^. To determine the test–retest reliability, the questionnaire was completed by 30 eligible women of reproductive age who had referred to the health centers of Tabriz city by random sampling method in two stages with a time interval of two weeks. Internal consistency was also assessed by determining Cronbach's alpha coefficient and Mcdonald’s omega coefficient for each factor and the whole instrument. Intra-class correlation coefficient (ICC) greater than 0.6 and Cronbach's alpha coefficient and Mcdonald’s omega coefficient above 0.7 were considered favorable^54^.

### Ethical consideration

The present study was approved by the Ethics Committee of Tabriz University of Medical Sciences (Ethics code: IR.TBZMED.REC.1400.1073). All ethical principles, including obtaining necessary permission from the main designers of the instrument (Baessler et al.), obtaining written informed consent from all participants, ensuring the confidentiality of their information, and freedom to withdraw from the study were observed at every step.

### Statistical analysis

SPSS Statistics 14 (IBM Corp, Armonk, NY, USA) and STATA 14 (Statcorp, college station, Texas, USA) and R software 4.2 (Psych package) were used for data analysis. In this study, the socio-demographic and obstetric characteristics, content validity, face validity, construct validity and reliability were determined respectively through Mean (SD) for quantitative variables and frequencies (percentages) for qualitative variables, CVR and CVI, Impact score, EFA and CFA and finally, Cronbach's alpha coefficient, McDonald's omega and ICC were assessed.

### Ethics approval and consent to participate

The current study was approved by the Ethics Committee of Tabriz University of Medical Sciences [ref: IR.TBZMED.REC.1400.1073]. Written informed consent to participate in the study was obtained from all the participants before enrolment. All methods were performed in accordance with the Declaration of Helsinki.

## Results

400 women of reproductive age with a mean (SD) age of 34.4 (7.2) (range 16–49) were included in this study between May 2022 and September 2022, with cluster sampling method. The mean (SD) of body mass index was 26.9 (4.1) and more than three quarters of them (82.3%) were housewives. Other socio-demographic and obstetric characteristics of the participants are summarized in Table 1.

**Table 1:** Socio-demographic and obstetric characteristics of participants for factor analysis of APFQ-IR (n = 400).

Characteristics	Mean	SD
Age (years)	34.4	7.2
BMI (kg/m^2^)	26.9	4.1

	Number	Percent
Gravidity		
< 3	299	74.8
≥ 3	101	25.3
Parity		
< 3	352	88.0
≥ 3	48	12.0
Education		
High school or below	286	71.5
College or above	114	28.5
Job		
Housewife	329	82.3
Employee	71	17.8
Income		
Not at all sufficient	74	18.5
Relatively sufficient	229	57.0
Completely sufficient	98	24.5
Smoking status		
Yes	3	0.7
No	397	99.3
Type of delivery		
NVD with episiotomy	125	32.9
NVD without episiotomy	13	3.4
C/S	210	55.3
Both	32	8.4
Hysterectomy		
Yes	4	1.0
No	396	99.0
Family history of PFDs		
Yes	17	4.2
No	383	95.8

*SD* standard deviation, *BMI* body mass index, *NVD* normal vaginal delivery, *C/S* ceasarean section.

The mean (SD) of the entire APFQ in the present study was equal to 0.72 (0.67), and for the 4 extracted factors including SF, BLF, BOF, and PS, it was 0.56 (0.77), 0.97 (1.06), 0.20 (0.66) and 1.16 (1.32) respectively (Table 1).

Assessing the content validity of the tool, CVI, CVR were obtained as 0.94 and 0.96, respectively, which indicates the good reliability of the instrument. But on the other hand, question 36 of the instrument (vaginal sensation during intercourse) (SF4) was corrected because it had a CVI = 0.58. Moreover, in the face validity review, all the items were described as appropriate and without ambiguity and difficulty and received a minimum score of 1.5. The details of the results of content and face validity assessment are shown in Table 2.

**Table 2:** The results for the content and face validity of the Iranian version of APFQ-IR (n = 10).

Item	CVI	CVR	Impact score
1. APFQ1	1.00	1.00	4.00
2. APFQ2	1.00	1.00	4.00
3. APFQ3	0.87	0.87	3.46
4. APFQ4	0.91	1.00	4.00
5. APFQ5	0.91	1.00	3.86
6. APFQ6	0.87	1.00	4.00
7. APFQ7	0.91	1.00	4.00
8. APFQ8	1.00	1.00	4.00
9. APFQ9	1.00	1.00	4.00
10. APFQ10	1.00	1.00	3.33
11. APFQ11	1.00	1.00	3.73
12. APFQ12	1.00	1.00	4.00
13. APFQ13	1.00	1.00	3.73
14. APFQ14	0.95	1.00	3.86
15. APFQ15	1.00	1.00	3.73
16. APFQ16	1.00	1.00	3.46
17. APFQ17	1.00	1.00	3.33
18. APFQ18	1.00	1.00	3.33
19. APFQ19	1.00	1.00	3.60
20. APFQ20	1.00	1.00	3.60
21. APFQ21	1.00	1.00	3.86
22. APFQ22	0.95	0.87	3.33
23. APFQ23	0.79	0.87	3.20
24. APFQ24	0.87	0.87	3.20
25. APFQ25	1.00	1.00	3.46
26. APFQ26	1.00	1.00	3.20
27. APFQ27	1.00	1.00	3.33
28. APFQ28	1.00	1.00	3.33
29. APFQ29	1.00	1.00	3.33
30. APFQ30	0.83	0.75	2.93
31. APFQ31	0.87	0.87	3.20
32. APFQ32	0.83	0.75	3.60
33. APFQ33	0.95	1.00	3.73
34. APFQ34	1.00	1.00	3.86
35. APFQ35	0.83	0.87	4.00
36. APFQ36	0.58	0.62	4.00
37. APFQ37	1.00	1.00	4.00
38. APFQ38	0.95	0.87	4.00
39. APFQ39	1.00	1.00	4.00
40. APFQ40	0.91	1.00	4.00
41. APFQ41	1.00	1.00	4.00
42. APFQ42	1.00	1.00	4.00
43. APFQ43	0.87	1.00	3.86

*CVI* content validity index, *CVR* content validity ratio.

**Figure 1:**
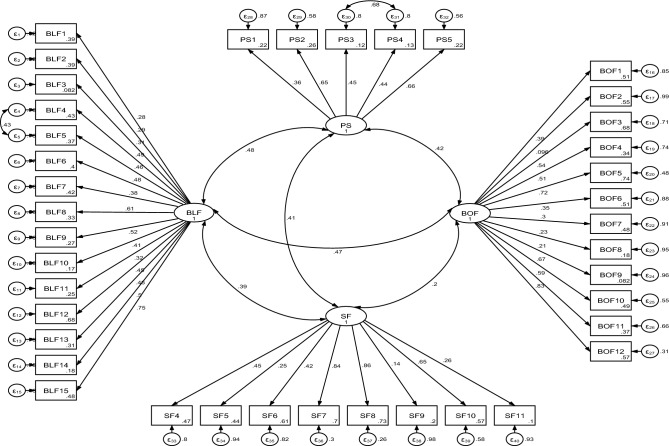
Factor structure model of the APFQ-IR based on CFA. (All factor loadings are significant at P < 0.001). *BLF* bladder function, *BOF* bowel function, *PS* prolapse symptom, *SF* sexual function.

In the construct validity assessment, the total number of questions in the original version of the instrument was 43, but due to the difference in the way of answering and missing (because only sexually inactive women had to answer them), 3 questions (SF1-SF3) were not included in the exploratory factor analysis. Finally, exploratory factor analysis was performed only on 40 items, which led to the extraction of 4 factors that explained 45.53% of the variance. During the process of exploratory factor analysis, questions 17 (BOF2), 24 (BOF9), 28 (PS1) and 41 (SF9) were also removed due to factor loading less than 0.3 and finally the number of questions was reduced from 43 to 36 questions (Fig. 1).

The four factors extracted during exploratory factor analysis are: The first factor was BLF, which includes 15 questions, accounting for 14.99% of the total variance. The second factor is BOF and has 10 questions, which explains 11.90% of the total variance. Finally, the third and fourth factors are PS, with 4 questions, and SF, with 7 questions, explaining 8.75% and 9.88% of the total variance, respectively (Table 3). The results indicating the adequacy of the sample size (Kaiser–Meyer–Olkin = 0.750) were obtained at a significance level of less than 0.001. Also, the result of Bartlett's sphericity test was significant, which indicated the acceptable performance of factor analysis according to the correlation matrix in the studied sample (P ≤ 0.001) (Table 4).

**Table 3:** Result of factor analysis of the APFQ-IR scale based on EFA (n = 400).

Scale item	Factors			
	1	2	3	4
Factor 1: BLADDER FUNCTION				
1. How many times do you pass urine in a day?	0.274			
2. How many times do you get up at night to pass urine?	0.279			
3. Do you wet the bed before you wake up at night?	0.282			
4. Do you need to rush/hurry to pass urine when you get the urge?	0.541			
5. Does urine leak when you rush or hurry to the toilet or can’t you make it in time?	0.530			
6. Do you leak with coughing, sneezing, laughing or exercising?	0.512			
7. Is your urinary stream (urine flow) weak, prolonged or slow?	0.352			
8. Do you have a feeling of incomplete bladder emptying?	0.606			
9. Do you need to strain to empty your bladder?	0.510			
10. Do you have to wear pads because of urinary leakage?	0.409			
11. Do you limit your fluid intake to decrease urinary leakage?	0.325			
12. Do you have frequent bladder infections?	0.413			
13. Do you have pain in your bladder or urethra when you empty your bladder?	0.422			
14. Does urine leakage affect your routine activities like recreation, socializing, sleeping, shopping etc.?	0.505			
15. How much does your bladder problem bother you?	0.761			
Factor 2: BOWEL FUNCTION				
16. How often do you usually open your bowels?		0.382		
17. Do you have to strain to empty your bowels?		0.538		
18. Do you use laxatives to empty your bowels?		0.510		
19. Do you feel constipated?		0.739		
20. When you get wind or flatus, can you control it, or does wind leak?		0.362		
21. Do you get an overwhelming sense of urgency to empty bowels?		0.288		
22. Do you leak watery stool when you don’t mean to?		0.227		
23. Do you have a feeling of incomplete bowel emptying?		0.647		
24. Do you use finger pressure to help empty your bowel?		0.587		
25. How much does your bowel problem bother you?		0.833		
Factor 3: PROLAPSE SYMPTOMS				
26. Do you experience vaginal pressure or heaviness or a dragging sensation?			0.450	
27. Do you have to push back your prolapse in order to void?			0.781	
28. Do you have to push back your prolapse to empty your bowels?			0.804	
29. How much does your prolapse bother you?			0.521	
Factor 4: SEXUAL FUNCTION				
30. During intercourse vaginal sensation is?				
31. Do you feel that your vagina is too loose or lax?				0.253
32. Do you feel that your vagina is too tight?				0.398
33. Do you experience pain with sexual intercourse?				0.820
34. Where does the pain during intercourse occur?				0.830
35. How much do these sexual issues bother you?				0.693
36. Other symptoms?				0.272
% of variance	14.99	11.90	8.75	9.88
Total score	45.53

**Table 4:** KMO and Bartlett's test of the Iranian version of APFQ-IR (n = 400).

Measures	Value
KMO measure of sampling adequacy	0.750
Bartlett’s test of sphericity approx	5417.0256
Df	780
P-value	< 0.001

*KMO* Kaiser–Meyer Olkin, *df* degree of freedom.

In confirmatory factor analysis (CFA), 4 factors obtained in exploratory factor analysis (36 items) were examined. The results showed that this model achieved a good level of fit, based on which the factorial structure can be confirmed (RMSEA = 0.08, SRMR = 0.08, TLI = 0.90, CFI = 0.93, *χ*^2^/df = 3.52) (Table 5).

**Table 5:** The model fit indicators of the APFQ-IR (n = 400).

Goodness of fit indices	CFA	Acceptable value
χ^2^	2576.843	
Df	732	
*χ*^2^/df	3.520	< 5
P-value	< 0.001	< 0.05
CFI	0.928	> 0.90
TLI	0.903	> 0.90
SRMR	0.089	< 0.10
RMSEA (90% CI)	0.079 (0.076–0.083)	< 0.08

*χ*^*2*^ Chi-square, *df* degrees of freedom, *χ*^*2*^*/df* normed Chi-square, *CFI* Comparative Fit Index, *TLI* Tucker–Lewis index, *SRMR* standardized root mean squared residual, *RMSEA* root mean square error of approximation.

Finally, to determine the reliability of the tool, (Cronbach's alpha = 0.85; McDonald's omega (95% CI) = 0.85 (0.83–0.87) and Intraclass Correlation Coefficient (95% CI) = 0.88 (0.74–0.94)), were obtained, showing a good instrument reliability. Moreover, ceiling effect was not observed in the overall value and sub-domains, but the floor effect in the overall score (APFQ-IR) was equal to 8.5% and the values of the sub-domains are detailed in Table 6.

**Table 6:** Reliability statistics, floor and celling effect of the APFQ-IR.

Factors	Cronbach’s α coefficient	McDonald's omega (95% CI)	ICC (95% CI) (n = 30)	Floor effect (%)	Celling effect (%)
BLF	0.78	0.79 (0.76–0.82)	0.90 (0.79–0.95)	33.3	0.0
BOF	0.77	0.80 (0.77–0.83)	0.83 (0.64–0.92)	23.8	0.0
PS	0.68	0.67 (0.60–0.74)	0.77 (0.52–0.89)	87.0	0.0
SF	0.71	0.76 (0.73–0.80)	0.95 (0.90–0.98)	35.3	0.0
APFQ (total)	0.85	0.85 (0.83–0.87)	0.88 (0.74–0.94)	8.5	0.0

*ICC* intra class correlation coefficient, *CI* confidence interval, *BLF* bladder function, *BOF* bowel function, *PS* prolapse symptom, *SF* sexual function, *APFQ* Australian Pelvic Floor Questionnaire.

## Discussion

Pelvic floor disorders (PFDs) are a significant health problem among women living in low and middle-income countries. This problem exists because many women with PFDs, due to misconceptions and lack of awareness of the existence of treatment options, fear of discrimination, feeling of shame and society's culture, hide their problem^55,56^. Therefore, the existence of a reliable instrument to measure the symptoms of PFDs, seems necessary. This study, aiming to psychometrically evaluate the APFQ among Iranian women, indicates that the Persian version of this questionnaire (APFQ-IR) has acceptable psychometric properties for evaluating PFDS, and can be used as a valid and reliable tool among Iranian women.

At the present time, despite the design of numerous questionnaires to evaluate PFDS with emphasis on the domains of urinary incontinence^57,58^, fecal incontinence^59^ and some for pelvic organ prolapse^60^, there are only a few valid questionnaires that cover all domains (bladder, bowel, prolapse and sexual domains), merged together. Among them, the ICI questionnaires (www.iciq.net), despite having strong criteria for assessment, are not designed to be used in clinical practice^61^.

Although the Pelvic Floor Distress Inventory-20 (PFDI-20) questionnaire, and the Pelvic Floor Impact questionnaire (PFIQ-7) are recommended by ICI with grade B^60,62^, these questionnaires (long and short form), are not designed to be used in routine urogenycological operations, since they are aimed at measuring the intensity and frequency of symptoms (never, occasionally, frequently, etc.), not at specifically evaluating sexual function. Therefore, these questionnares, alone are not useful in clinical practice. The only questionnaire that integrates all areas is The Global Pelvic Floor Bother Questionnaire, which has 9 questions, but one of its disadvantages is the lack of dedicated sections for each area and the allocation of only one question (question 9) to sexual activity^63^.

During the process of exploratory factor analysis in this study, 4 factors were extracted for 36 questions of the questionnaire, including BLF, BOF, PS and SF, which explained about 45.53% of the variance. As a part of the instrument’s validity assessment, the value of KMO and the significance of Bartlett's test was also assessed which confirmed the adequacy of the model. Although the psychometric properties of APFQ have been investigated in several languages worldwide, the construct validity has only been examined in Arabic and Spanish versions. The first study was conducted on the Arabic version (2021) by Malaekah et al., who identified four factors explaining 36.64% of the variance. The results of exploratory analysis showed KMO = 0.806 and Bartlett sphericity test = 4150.46^64^. The second study was performed on the Spanish version (2022) by Molina-Torres et al., who identified two factors explaining 31.26% of the variance. The results of exploratory factor analysis showed KMO = 0.858, with a significant value in the Bartlett sphericity test (P < 0.001)^37^. The factors extracted during exploratory factor analysis are parallel and in line with the factors reported in Pelvic Floor Distress Inventory-20 (PFDI-20) and Pelvic Floor Impact Questionnaire (PFIQ-7) measures^62^, with the difference that the APFQ questionnaire, having a sexual performance section, is more complete than the two questionnaires mentioned.

Despite the ICC's emphasis on the use of these two questionnaires, APFQ has some advantages over them. The advantage and strength of the APFQ questionnaire compared to other questionnaires is the existence of a separate section for examining women's sexual performance, the answering method which focuses on measuring the frequency and severity of symptoms, level of bother and issues related to the quality of life in these special conditions.

In order to determine internal consistency, Cronbach's alpha coefficient for the APFQ-IR and its range for the subscales were obtained as 0.85, 0.68–0.78, which in the reported values for these parameters in original scale were 0.74–1.00, almost within the range of the original scale^33^. The obtained results were also similar to the Turkish version (0.73–0.86)^35^, and Serbian (0.82–0.84)^36^, but compared to the Spanish version (0.83–0.93)^37^ and Chinese (0.83–0.89)^34^ were smaller. Moreover, to determine the reliability of this study, the ICC was obtained 0.77–0.95, which is in accordance with the range of the original instrument (0.74–1.00)^33^ and higher than the Chinese version (0.22–0.88)^34^ and the Spanish version (0.59–0.96)^37^.

Because of the strong role of women in the family’s and society’s health, the high prevalence of the PFDs and the wide range of complications caused by this situation is considered as a silent alarm about reducing the QOL and interfering with the roles of women in society. As a result, the importance of a dedicated instrument to evaluate the symptoms of PFDs by health care providers in health centers, and subsequently, training, diagnostics and therapeutic measures, becomes more prominent.

### Strength and limitation

Some of the strengths of this study are: Investigating the psychometric properties of APFQ-IR for the first time in Iran, the comprehensiveness of the tool and coverage of all areas of PFDs, especially the sexual function section, the way of responding with an emphasis on measuring the severity and frequency of symptoms compared to other tools in this field, using the combination of the dual panel method and the five stages method for the purpose of the translation process and finally providing the possibility of comparison with other versions from other countries. Using the same set of data in order to perform exploratory and confirmatory factor analysis, not including general quality of life questions in the questionnaire, and the smaller number of women respondents under the sexual function scale due to sexual inactivity are the limitations of the present study.

## Conclusion

The Persian version of the questionnaire, APFQ-IR, has a good validity and reliability and has acceptable psychometric properties, thus can be used both for research purposes and for clinical evaluation of PFDs symptoms in health centers. Thus, health policy makers should do their best to design special programs for screening, training, diagnosis and treatment measures in order to evaluate and follow up women with PFDs with the aim of improving their performance and QOL.

## Data Availability

The datasets generated and/or analyzed during the current study are not publicly available due to the limitations of ethical approval involving the patient data and anonymity, but are available from the corresponding author upon reasonable requests.

## Abbreviations

PFDs: Pelvic floor disorders
APFQ: Australian Pelvic Floor Questionnaire
QOL: Quality of life
POP: Pelvic organ prolapse
PROMs: Patient report outcomes
EFA: Exploratory factor analysis
CFA: Confirmatory factor analysis
ICC: Intra-class correlation coefficient
SD: Standard deviation
CVI: Content validity index
CVR: Content validity ratio
df: Degree of freedom
KMO: Kaiser–Meyer–Olkin
RMSEA: Root mean squared error of approximation
CFI: Comparative fit index
TLI: Tucker–Lewis index
